# Black Sesame Pigment Ameliorates Non-Alcoholic Fatty Liver Disease via Modulation of the Gut–Liver Axis and HIF-1 Signaling Pathway

**DOI:** 10.3390/antiox15020177

**Published:** 2026-01-30

**Authors:** Qian Huang, Zhuowen Liang, Qingpeng Li, Ke Wang, Shuang Zhu, Wei Xiao, Lin Zhou

**Affiliations:** 1 Guangdong Provincial Key Laboratory of Pharmaceutical Preparations Research and Evaluation, Guangdong Pharmaceutical University, Guangzhou 510006, China; 1112342004@stu.gdpu.edu.cn (Q.H.); 2112348109@stu.gdpu.edu.cn (Q.L.); 2112348092@stu.gdpu.edu.cn (K.W.); zhushuang@gdpu.edu.cn (S.Z.); 2 Key Laboratory of Glucolipid Metabolic Disorder, Ministry of Education, Guangdong Pharmaceutical University, Guangzhou 510006, China; 2112342050@stu.gdpu.edu.cn

**Keywords:** black sesame pigment, nonalcoholic fatty liver disease, gut–liver axis, liver organoids, HIF-1 signaling pathway

## Abstract

Black sesame pigment (BSP), a key macromolecular component of the traditional food–medicine black sesame, holds potential for improving non-alcoholic fatty liver disease (NAFLD), but its mechanisms remain unclear. We evaluated BSP and fired black sesame pigment (FBSP) in a high-fat diet/streptozotocin-induced NAFLD mouse model. An integrated multi-omics strategy—encompassing network pharmacology, urinary metabolomics, and 16S rRNA sequencing—was employed to identify potential targets and pathways. Key findings were subsequently validated in a human liver organoid model of NAFLD. FBSP treatment significantly alleviated hepatic steatosis and dysfunction in mice. Multi-omics analysis revealed that FBSP reshaped the gut microbiota (increasing Lactobacillus and Bacteroides) and influenced host glycolysis/gluconeogenesis metabolism. Both omics predictions converged on the HIF-1 signaling pathway. In human liver organoids, FBSP reduced lipid accumulation and inflammation, and modulated the expression of core HIF-1 pathway genes. This study demonstrates that FBSP ameliorates NAFLD, potentially through a gut–liver axis mechanism that involves microbiota remodeling and subsequent modulation of the hepatic HIF-1 signaling pathway. Our findings position FBSP as a promising food-derived candidate for NAFLD intervention.

## 1. Introduction

Non-alcoholic fatty liver disease (NAFLD) is a progressive disease associated with multiple factors, including high-fat diet and insulin resistance, characterized primarily by fat accumulation in hepatocytes as its main feature of a clinicopathological syndrome. Additionally, NAFLD is closely related to metabolic syndrome and type 2 diabetes, among other diseases. Its progression includes stages such as non-alcoholic fatty liver (NAFL), non-alcoholic steatohepatitis (NASH), cirrhosis, and hepatocellular carcinoma (HCC) [1,2]. The U.S. Food and Drug Administration approved Rezdiffra, an oral small-molecule drug, for the treatment of NAFLD. Meanwhile, in the domains of food and medicine health, the discovery and application of useful medications and food homologous substances is a promising approach for the treatment and management of NAFLD.

Sesame is a major edible oilseed crop worldwide, characterized by its high oil content and significant economic potential. Notably, its planting area accounts for more than 90% of the global total. Among its varieties, due to its unique nutritional and health benefits, black sesame (BS) was officially included in the “food and medicine homology” list by the Ministry of Health on 28 February 2002 and has been increasingly attracting attention from the public. Additionally, black sesame pigment (BSP), an important by-product of black sesame, serves as an effective natural source of bioactive pigments. It can be utilized as a coloring agent and functional ingredient in the food industry, holding considerable economic and industrial value. The expanding application of medicinal and edible ingredients in functional foods meets consumer health demands and circumvents the adverse effects typically linked to medications. A comprehensive understanding of the phytochemical constituents and bioactivities of black sesame can promote its high-value utilization.

BS is a traditional tonic food containing a variety of biologically active substances such as polysaccharides, pigments, and lignans, which has antioxidant activity and protects against liver damage. We previously identified phytochemicals with antioxidant effects in different varieties of sesame seeds, though their mechanisms of action remain unclear [3,4]. Meanwhile, the in vitro antioxidant activity of BS has been confirmed, but the biological activity of BS has not been sufficiently validated in human studies [5]. Over the past several years, a substantial body of evidence has been accumulated supporting the crucial role of the gut–liver axis in NAFLD [6,7]. Hence, manipulating metabolites and gut microbiota composition could be a promising therapeutic approach for NAFLD. Recently, studies have revealed regulating metabolic disorders improves NAFLD progression [8,9]. Multi-omics technologies have a wide range of applications in the study of metabolic diseases, including diseases such as diabetes, obesity, and NAFLD [10,11]. Our recent study has found that BSP exhibits significant antioxidant activity and therapeutic effects on NAFLD. Since most studies have focused on the changes in chemical composition and antioxidant activity of phenolic small molecules in sesame [12,13,14], the activity of BSP macromolecules was severely underestimated. Given these findings, we therefore hypothesized that BSP, a key active component of BS, ameliorates NAFLD by regulating the HIF-1 signaling pathway.

This study investigated the beneficial effects of BSP against NAFLD at the animal level. Furthermore, the new mechanism insights were investigated by network pharmacology, urinary metabolomics, and intestinal flora genomics to explore the effectiveness of BSP against NAFLD and further explore its molecular mechanism using liver organoids model [15]. The mechanism targets of the HIF-1 signaling pathway was analyzed. This study aims to elucidate the protective mechanism of BSP in NAFLD and provide a basis for the development of functional foods for liver injury and other dietary supplements.

## 2. Materials and Methods

### 2.1. Extraction and In Vitro Digestion of BSP and FBSP

BS was purchased from Shandong Zaozhuang Grain Warehouse Four Seasons Co., Ltd. (Zaozhuang, China). Fired black sesame (FBS) was obtained by subjecting black sesame seeds to a roasting process. An appropriate amount of black sesame seeds was placed in a heating machine for roasting. The roasting temperature was set at 220 °C, and the roasting time was 30 min. The roasting process was completed when popping sounds were heard, the black sesame seeds were slightly puffed, or surface cracking appeared, accompanied by the release of aroma. The extraction method of BSP was referenced from previous studies, utilizing the alkali solubilization and acid precipitation method to obtain BSP, with reference to “Chinese pharmacopoeia” (2020 version) to prepare the BS and fired black sesame pigment (FBSP) extraction. The NMR-^1^H spectrum measurements and scanning electron microscopy were conducted by Shenzhen Tsinghua University Research Institute. The in vitro simulated gastrointestinal digestion model included oral, gastric, small intestinal, and colonic phases.

### 2.2. Determination of Yield and Color Value

The calculation formula for the extraction yield of black sesame pigment before and after frying is as follows: D = m × W/100. D represents the extraction yield (%), m is the mass of extracted pigment (g), W is the mass of black sesame seeds (g).

An appropriate amount of pigment was taken, and 0.05 mol/L sodium bicarbonate solution was added to prepare a solution of a certain concentration. Using 0.05 mol/L sodium bicarbonate solution as a blank, UV-vis absorption spectra were scanned in the range of 190–800 nm, and the wavelength corresponding to the maximum absorption (λmax) was recorded. Exactly 0.1000 g of pigment was accurately weighed, and an appropriate amount of 0.05 mol/L sodium bicarbonate solution was added and stirred. The mixture was dissolved with the assistance of a 60 °C water bath. The solution was then diluted to 100 mL using 0.05 mol/L sodium bicarbonate solution. After thorough mixing, precisely 4 mL of the pigment solution was removed, and the volume was adjusted to 100 mL using 0.05 mol/L sodium bicarbonate solution. The absorbance of the diluted pigment solution was measured at the maximum absorption wavelength, and the color strength (E) was calculated using the formula: E = A/(100 × C). E represents the absorbance value measured at the maximum absorption wavelength when the concentration of the test solution is 1% (using a 1 cm cuvette), A is the actual absorbance of the test solution, C is the concentration of the test solution (g/mL).

### 2.3. Characterizations of Pigments

The samples were dissolved in sodium bicarbonate (0.05 M) to a final concentration of 1 mg/mL and their absorption properties were determined using UV-vis spectroscopy. A Nicolet VERTEX 33 spectrometer (Bruker, Ettlingen, Germany) was used to analyze the samples using FT-IR at wave numbers 4000–400 cm^−1^.

### 2.4. ^1^H NMR Spectroscopy Analysis

^1^H NMR spectroscopy of the purified black sesame pigment was performed on a Bruker Avance NEO 600 MHz NMR spectrometer equipped with a cryogenic probe. Approximately 2–3 mg of the sample was dissolved in 0.6 mL of dimethyl sulfoxide-d_6_ (DMSO-d_6_), vortexed thoroughly, and transferred into a standard 5 mm NMR tube for measurement.

The spectrum was acquired using a standard single-pulse sequence (zg30). Acquisition parameters were set as follows: spectral width 11,904.763 Hz (=19.84 ppm); acquisition time 2.75 s; relaxation delay 1.0 s; pulse width 10.00 μs; number of scans 8; and data points 65,536. The experiment was conducted at 298.1 K (25 °C).

Data were processed with Bruker Topspin software (version 4.5.0). The free induction decay was Fourier-transformed after applying a 0.30 Hz line-broadening window function. Chemical shifts (δ) are reported in ppm relative to the residual solvent signal of DSO-d_5_ (δ 2.50 ppm).

### 2.5. Field Emission Scanning Electron Microscopy Analysis

The micromorphology of the black sesame pigment sample was observed using a field emission scanning electron microscope (FE-SEM). The specific procedures were as follows: a small amount of the freeze-dried pigment powder was uniformly dispersed onto the surface of conductive tape adhered to an aluminum sample stub, and loosely attached particles were removed using a rubber blow bulb. Subsequently, a layer of platinum-gold film approximately 10 nm thick was sputter-coated onto the sample surface using an ion sputtering coater to enhance its conductivity. Observations were conducted using a MIRA3 TESCAN field emission scanning electron microscope (TESCAN, Shanghai, China). The testing conditions were as follows: the accelerating voltage was set at 5.0 kV, and imaging was performed using a secondary electron detector. The working distance during observation was 11.85 mm, and the magnification was set at 2.00 k× and 5.00 k×, corresponding to a field of view width of approximately 126 μm.

### 2.6. In Vitro Simulated Digestion Methods

Oral digestion step: 2 g of black sesame pigment sample was weighed out, and 20 mL of α-amylase/calcium chloride solution (25 μL/mL, pH 7) was added. The mixture was incubated at 37 °C with shaking at 120 rpm for 10 min.

Gastric digestion step: The oral digestion product was adjusted to pH 2 using 6 mol/L HCl. Then, 10 mL of artificial gastric juice was added and mixed well (prepared by taking 1.64 mL of dilute HCl, adding approximately 80 mL of water, and mixing with 1 g of pepsin, followed by dilution to 100 mL with water). The mixture was incubated at 37 °C with shaking at 120 rpm for 1 h.

Small intestinal digestion step: The gastric digestion product was adjusted to pH 6 by adding 0.9 mol/L NaHCO_3_ solution. Then, 10 mL of artificial intestinal juice was added (prepared by dissolving 0.68 g of KH_2_PO_4_ in 50 mL of water, adjusting the pH to 6.8 with 0.1 mol/L NaOH solution, and mixing with 1 g of pancreatin dissolved in a suitable amount of water. The two solutions were mixed well and diluted to 100 mL with water). The mixture was incubated at 37 °C with shaking at 120 rpm for 2 h.

Large intestinal digestion step: The small intestinal digestion product was adjusted to pH 4 using 6 mol/L HCl. Then, 10 mL of artificial colon juice was added (prepared by dissolving 5.59 g of K_2_HPO_4_ and 0.41 g of KH_2_PO_4_ in water to a total volume of 1000 mL). The mixture was incubated at 37 °C with shaking at 120 rpm for 14 h.

### 2.7. Animal Experiments

NAFLD model mice were induced by using a high-fat diet (HFD) combined with streptozotocin (STZ) [16]. Eight-week-old male Kunming mice were housed in an SPF-grade environment (N = 6). All animal experiments were approved by the Animal Care and Use Professional Committee of Guangdong Pharmaceutical University (Approval No. GDPULAC2022076. Animal Approval Date: 3 March 2022) and strictly followed the National Institutes of Health Guide for the Care and Use of Laboratory Animals. After 3 days of acclimatization, the HFD/STZ-induced NAFLD mouse model was established. The naive control (NC) group mice were continuously fed a standard diet, while the remaining mice were fed an HFD (24% fat, 24% protein, and 41% carbohydrates). In the high-fat diet, fat provides approximately 45% of the total energy. The HFD groups received intraperitoneal injections of STZ daily for 1 day and were fed an HFD for 4 weeks. Mice with fasting blood glucose (FBG) ≥11.1 mmol/L were selected as successful modeled type 2 diabetic mice (T2DM). Hematoxylin-eosin (H&E) staining was performed for histopathological examination of liver tissue, and the liver index was comprehensively evaluated. The levels of serum alanine aminotransferase (ALT) and aspartate aminotransferase (AST), as well as the concentrations of total cholesterol (TC) and triglycerides (TG) in plasma, were measured to assess liver injury in mice with non-alcoholic fatty liver disease.

Drug treatment was administered after 4 weeks of HFD feeding. Drugs were administered intragastrically once daily for 4 weeks, including metformin group (PC group) (300 mg/kg); BSP-L (200 mg/kg); BSP-H (800 mg/kg); FBSP-L (200 mg/kg); and FBSP-H (800 mg/kg). Saline was administered intragastrically once daily for 4 weeks in the NC group and NAFLD model group (DC group). At the end of the experiment, urine and feces were collected; serum and tissue samples were obtained for subsequent analysis.

### 2.8. Fasting Blood Glucose

The fasting blood glucose values of mice in each group were measured on days 0, 14, and 28 after administration. Prior to blood glucose measurement, the mice were fasted for 12 h with free access to water. Blood glucose levels were measured using a blood glucose meter with blood collected from the tail vein using a blood glucose test strip.

### 2.9. Liver Histology Examination

Mouse liver tissues were dehydrated and paraffin embedded. Serial sections 3–5 μm thick were cut from each block, placed on glass slides, and stained with hematoxylin and eosin (H&E). Histopathological changes were described and scored (steatosis, lobular inflammation, hepatocellular ballooning, and fibrosis), using semi-quantitative grading of five grades (0–4), taking into consideration the severity of the changes. A generic grading criterion was used: Zero 0 = no lesion; 1 = minimal change; 2 = mild change; 3 = moderate change; and 4 = marked change [17,18].

### 2.10. Determination of Plasma Biochemical Parameters

A biochemistry analyzer was used to measure liver function-related parameters in the plasma of mice in each group, including the activities of alanine aminotransferase, aspartate aminotransferase, total cholesterol, and triglycerides.

### 2.11. Network Pharmacology Analysis

Black sesame effective compounds were collected from the Traditional Chinese Medicine Systems Pharmacology (TCMSP) database using the ADME filter method, and the main parameters included oral bioavailability (OB) and drug-likeness (DL) [19]. Target genes associated with liver injury were acquired from the Genecards database. The liver injury targets were compared with the predicted black sesame targets, and a Venn diagram was made with Venny 2.1.0. These common target proteins were selected to act on the STRING platform to construct a protein–protein interaction (PPI) network model. Module analysis based on Cytoscape’s Molecular Complex Detection (MCODE) plugin was performed on the PPI network to screen scoring modules. Biological processes involved were analyzed by Gene Ontology (GO) and Kyoto Encyclopedia of Genes and Genomes (KEGG) pathway enrichment analysis.

### 2.12. LC-MS Metabolomics Analysis

Urine metabolomics analysis was conducted by Meiji Biotech Co., Ltd. Urinary metabolites were determined using liquid chromatography-tandem mass spectrometry (LC-MS) [20]. SIMCA-P 14.1 software (Umetrics, Malmö, Sweden) was used to conduct principal components analysis (PCA), partial least squares analysis discriminant analysis (PLS-DA), and orthogonal partial least squares (OPLS-DA) analysis of the normalized data. The OPLS-DA model was constructed based on internal sevenfold cross-validation. The R2 and Q2 ranged from 0 to 1, where 1 indicates perfect fitness and predictive ability. Variable importance of projection (VIP) values was used to characterize the contributions of the metabolites to the various Y change rates in OPLS-DA. VIP > 1 of the metabolites indicated that they markedly contributed to group classifications. Meanwhile, an independent sample *t*-test was used to validate the major contributing variables from the OPLS-DA models. A value of *p* < 0.05 was considered statistically significant. Based on the VIP values (VIP > 1), *t*-test (*p* < 0.05), and fold change (FC < 0.8 or >1.2), the differential metabolites were selected between the control group and the model group and identified. KEGG was used to analyze the differential metabolic pathways and to obtain the related enzymes. These enzymes were then selected to act on the STRING platform to construct a PPI network model with the key targets of network pharmacology.

### 2.13. Gut Microbiota Analysis

Fecal 16S rRNA sequencing analysis was conducted by Meiji Biotech Co., Ltd. (Shanghai, China). Genomic DNA was extracted from the fecal samples and quantified [20]. Microbial community composition was assessed using 16S metagenomic analysis, Alpha diversities (Good’s coverage, Shannon, Chao1, observed species, and PD Whole Tree), and beta diversities were estimated using q2-diversity according to the minimum number of sequences in the samples. Kruskal–Wallis’s rank-sum tests were used to detect significant differences in alpha diversity. Beta diversities were visualized using PCA and principal coordinates analysis (PCoA) plots. Based on the absolute abundance of OTUs and annotation information, the proportions of the sequence count at each taxonomic level (kingdom, phylum, class, order, family, genus, and species) for each sample relative to the total sequence count were calculated to identify differences in gut microbiota among different groups. The Phylogenetic Investigation of Communities by Reconstruction of Unobserved States (PICRUST) 2.0 pipeline was used to predict the potential functions of the bacterial communities based on 16S rRNA sequences.

### 2.14. Human Liver Organoid Culture

Liver organoids were obtained from Orgen BioTech Company (Beijing, China) [21,22] and cultured in a medium supplemented with 600 μM oleic acid and palmitic acid (free fatty acids, FFAs) to establish a non-alcoholic fatty liver model (AdDMEM/F12 supplemented with 2 mM glutamine, 10 mM Hepes, 1 × N2, 1 × B27, 50 ng/mL EGF, 25 ng/mL HGF, 100 ng/mL FGF10, l10 nM gastrin, 10 μM forskolin, 5 μM A8301, 10 mM nicotinamide, 1.25 mM N-acetylcysteine, 10 μM Y-27632, 100 ng/mL R-SPondin1, 100 ng/mL noggin, and 1% penicillin–streptomycin solution) [23,24,25]. The NC groups remained in a normal medium without FFAs. Then, organoids were treated with 150 μg/mL docetaxel phosphocholine or 5 mg/mL BSP digestion products.

### 2.15. Histology and Immunofluorescence Analysis

Organoid cultures were fixed overnight in 4% formalin at room temperature. Paraffin-embedded organoids were serially sectioned into 10 μM slices. These were deparaffinized and rehydrated, followed by staining for hematoxylin and eosin (HE) or antigen retrieval in sub-boiling 10 mM sodium citrate buffer (pH 7.4) for 10 min. For IF, slides were permeabilized in 0.5% Triton X-100 (Macklin, Shanghai, China) in PBS and blocked in 1% goat serum in PBS for 30 min at room temperature. After blocking, the slides were incubated with anti-ALB (Proteintech, Rosemont, IL, USA, 16475-1-AP, 1:250 for IF) or anti-AFP (Proteintech, 14550-1-AP, 1:250 for IF) overnight in a humidified chamber at 4 °C. Sections were washed with PBS (three times for 5 min each) and incubated with Alexa Fluor secondary antibodies (Proteintech, CTK0101, 1:400 for IF) for 1 h. After washing with PBS, the slides were mounted with 4,6-diamidino-2-phenylindole. Images were acquired with an inverted microscope.

### 2.16. Oil Red Staining and Measurement of Lipid Accumulation

The cryosections of the liver organoids with or without FFAs exposure were washed three times with PBS. To assess liquid droplet formation, the samples were stained with Oil Red O (GP1067, Servicebio, Wuhan, China). Images were obtained with a fluorescence microscope. The intracellular triglyceride and total cholesterol accumulation in liver organoids with FFAs exposure were measured using the triglyceride assay kit according to the manufacturer’s instructions.

### 2.17. Quantitative Real-Time PCR

Total mRNA was isolated from liver organoids using TRIzol reagent. The final mRNA concentration was adjusted to 500 ng/μL. The RNA concentration and quality were determined using a nanodrop. The cDNA was produced from 1 μg RNA and then amplified using Ex Tap DNA polymerase (K1036, APExBIO, Shanghai, China) under the following reaction conditions: denaturation at 95 °C for 15 s, annealing at 60 °C for 30 s, and extension at 95 °C for 1 min, with a cycle number of 40. Gene expression levels were calculated using the ΔΔC_t_ method, with GAPDH RNA serving as the internal reference. The sequences of all primers used are provided in Table S1.

### 2.18. Statistical Analysis

Statistical analysis was performed using GraphPad Prism software (version 12.0.0). All data are presented as mean ± SEM and were compared using 2-tailed unpaired Student’s *t* test or one-way analysis of variance (ANOVA) with Tukey post hoc tests. Statistical significance was defined as * *p* < 0.05, ** *p* < 0.01, and *** *p* < 0.001, with ns indicating no significant difference.

## 3. Results

### 3.1. Extraction and Structural Characterization of Pigment

**Figure 1:**
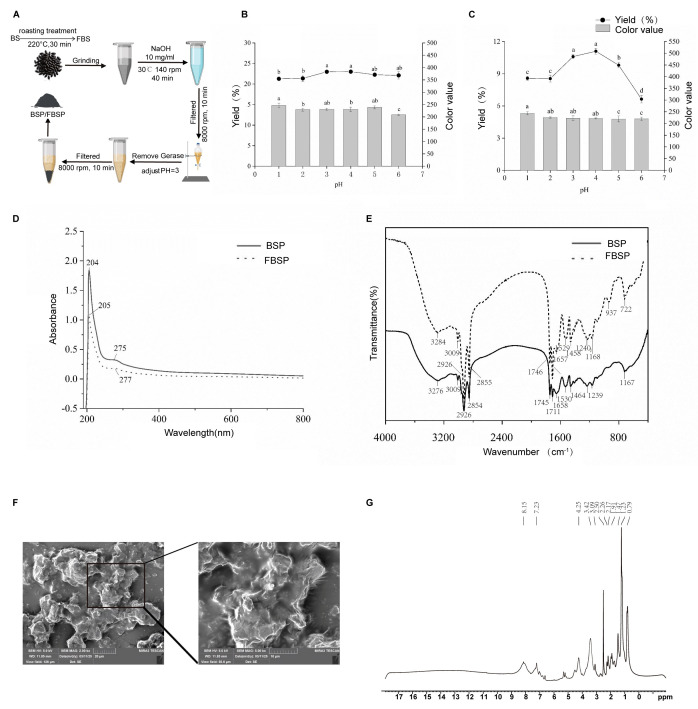
Extraction and structural characterization of BSP and FBSP. (**A**) Extraction process of BSP and FBSP. (**B**,**C**) Effect of varying acidic pH on BSP (**B**) and FBSP (**C**), different letters indicate significant differences (*p *< 0.05). (**D**) UV-vis spectra of BSP and FBSP. (**E**) FT-IR spectra of BSP and FBSP. (**F**) Scanning electron microscopy images of FBSP (Scale bar = 20 µm). (**G**) NMR-^1^H spectrum of FBSP.

The BSP and FBSP extraction processes are shown in Figure 1A. During the pigment extraction process, the optimal pH for the acid precipitation endpoint was selected based on the pigment yield and color value as reference standards. The effects of different acid precipitation pH on the pigment yield and color value of BSP and FBSP are shown in Figure 1B,C. Considering both the pigment yield and color value, the optimal acid precipitation endpoint pH for BSP and FBSP was selected as 3.

Additionally, structural characterization was performed on the BSP and FBSP pigments. The UV-Vis spectra of BSP and FBSP exhibited similar peak shapes (Figure 1D), with maximum absorption wavelengths of 204 nm and 205 nm, respectively, which is consistent with previous reports on melanin UV-Vis spectra [26]. When the wavelength exceeded 210 nm, the absorbance decreased with increasing absorption wavelength, which is consistent with the typical UV-resistant function of melanin reported previously [27]. A small absorption peak in the 270–280 nm range indicated the presence of conjugated structures in the extracted pigments [28]. Additionally, in the 400–800 nm region, the logarithm of absorbance exhibited a linear curve with a negative slope against the absorption wavelength, which is another key characteristic of melanin [29]. Furthermore, in the Fourier transform infrared (FT-IR) spectral characterization (Figure 1E), the typical infrared characteristic peaks of the BSP and FBSP were consistent with those of melanin [30,31,32]. These results confirmed that the extracted pigments were melanin. The melanin particles, as illustrated in the micrographs, primarily exist as individual entities, though some have formed aggregated clusters, and exhibit irregular shapes with dimensions ranging from 10 to 20 μm. Their surface texture appears rough, and layered structures are evident. The peripheries of the particles are distinctly visible in the images, with no apparent pores or cracks on their surfaces (Figure 1F). Aromatic protons typically resonate between 6–9 ppm, a distinctive feature of melanin. Therefore, the peaks at 7.23 ppm and 8.15 ppm are likely characteristic signals for melanin. These absorption peaks align with the molecular structure of melanin, particularly the presence of aromatic rings, hydroxyl, and amide functional groups. Notably, hydroxyl and amide protons are integral components of melanin structures, suggesting that the peaks at 3.42 ppm and 4.25 ppm may serve as characteristic signals. While methyl and methyl-adjacent protons are common in many organic compounds, these peaks may also act as diagnostic signals if multiple methyl groups are present in the melanin structure (Figure 1G).

### 3.2. The Protective Effects of Pigment on NAFLD

**Figure 2:**
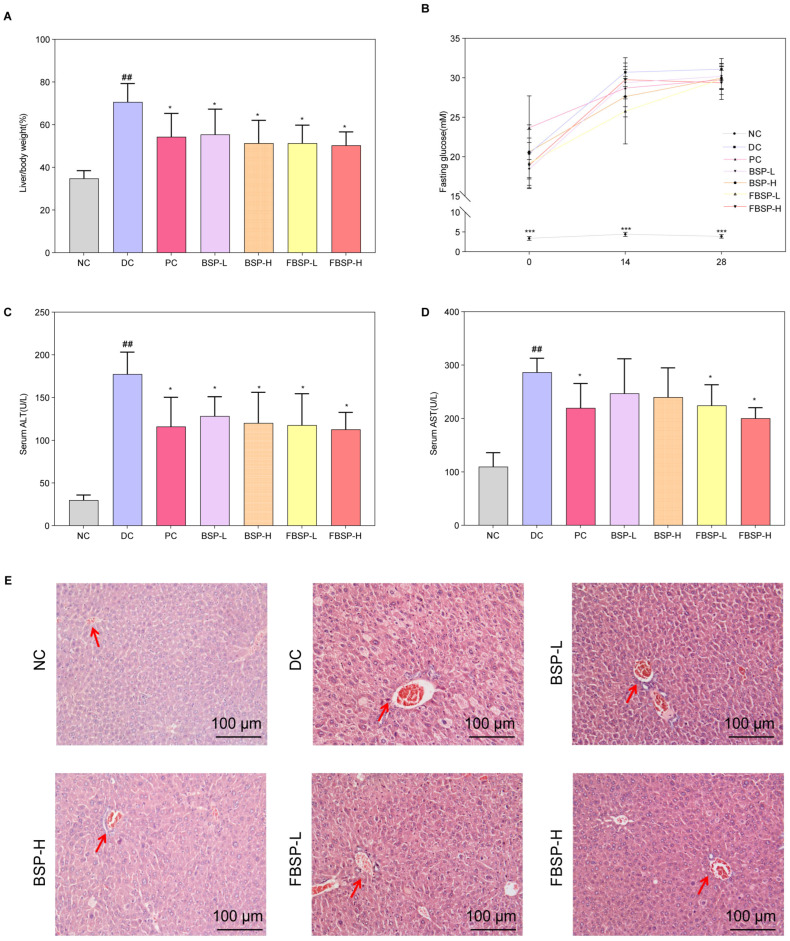
The protective effects of pigment on NAFLD. (**A**) The liver/body weight comparison. (**B**) Fasting glucose. (**C**) ALT level (U/L). (**D**) AST level (U/L). (**E**) H&E staining of the liver, and the representative areas were highlighted by arrows (scale bar = 100 µm). (## *p* < 0.01 compared with NC group, * *p* < 0.05, *** *p* < 0.001 compared with DC group).

To validate the protective effect of BSP and FBSP against NAFLD, we established a mouse model of NAFLD by inducing it with a high-fat diet combined with streptozotocin (HFD + STZ). Compared with the NC group, the organ index of the DC group mice significantly increased, and after BSP and FBSP treatment, the organ index significantly decreased (*p* < 0.05) (Figure 2A). Additionally, in the model group mice, fasting blood glucose, serum ALT, and AST levels were significantly increased, and after drug intervention, the fasting blood glucose (Figure 2B), serum ALT (Figure 2C), and AST (Figure 2D) levels in the treatment groups were significantly reduced (*p* < 0.05). Furthermore, the plasma TC and TG contents in the model group mice were significantly increased (Figure S1), and after BSP and FBSP intervention, the contents in each group were significantly reduced (*p* < 0.05).

H&E staining results showed that in the NC group, the morphology of hepatocytes was clearly visible, the hepatic lobular structure was intact without any pathological changes, and fat accumulation was absent in the cytoplasm (Figure 2E). In NAFLD mice, the lobular structure was present, but the sinusoidal structure was slightly disorganized, with partial hepatic steatosis, scattered hepatocyte swelling, and focal point-like necrosis within the lobule, accompanied by mild inflammatory cell infiltration in local areas. Significant fatty changes were observed in hepatocytes, with numerous fat droplets accumulated in the cytoplasm. After treatment with BSP and FBSP, H&E-stained sections revealed mild hepatocyte swelling around the portal vein and central vein areas, minimal fibrotic tissue growth, and minimal lymphocytic infiltration. Although a small amount of cellular edema was still observable under microscopy, nearly no cell death was observed, and the number of fat droplets was reduced to varying degrees. This indicates that different doses of BSP and FBSP resulted in varying degrees of improvement in liver structure in mice. Particularly, the FBSP-H group showed hepatocyte morphology closer to that of the NC group, with clearer lobular and sinusoidal structures. The histopathological score is shown in Figure S2. Combining liver index, liver function results, and hepatic H&E pathological section results, it can be seen that BSP and FBSP can improve both functional and structural liver abnormalities in NAFLD mice, among which, the hepatoprotective effect of FBSP was superior to that of BSP. This may be attributed to the alteration of pigment structure by roasting, such as glycosidic bond cleavage, leading to the formation of more stable and reactive structures, thereby enhancing antioxidant capacity.

### 3.3. Network Pharmacology Analysis

#### 3.3.1. Target Identification of BSP for NAFLD

**Figure 3:**
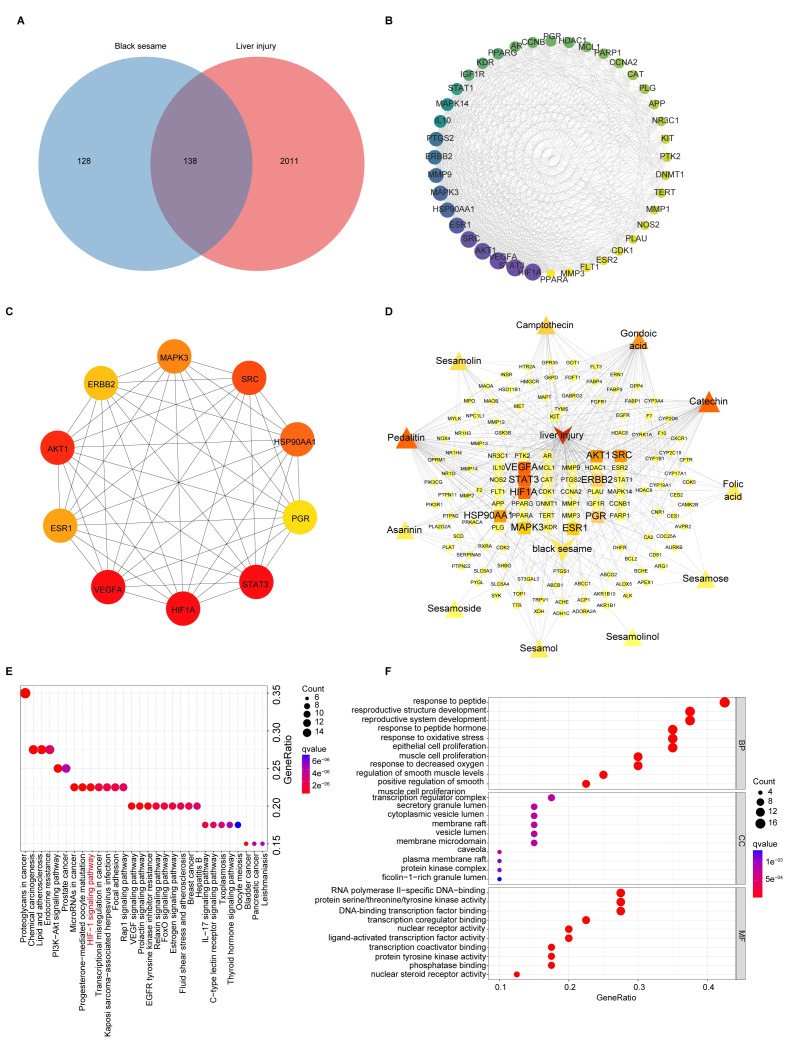
Analyses of targets and pathways that BSP improved in NAFLD by network pharmacology. (**A**) Venn diagram of BS targets and liver injury-related targets. (**B**) Cluster 1 of the Protein-protein interaction (PPI) network based on MCODE analysis. The area and color depth of nodes was proportional to the degree value of targets. (**C**) Top 10 core targets of 138 targets ranked by MCC method with cytoHubba plugin. The color depth of nodes was proportional to the degree value of targets. The color depth of nodes was proportional to the degree value of targets. (**D**) Relationships between drug, compound, targets and disease of BS, for the treatment of liver injury. The area and color depth of nodes was proportional to the degree value of targets. (**E**) KEGG analysis of the therapeutic target genes of BS for the treatment of liver injury. (**F**) GO (BP, MF, CC) analysis of the therapeutic target genes of BS for treatment of liver injury.

11 bioactive compounds of BS were obtained from the TCM-SP database, corresponding to 266 target genes (Table S2). A total of 8332 liver injury targets were obtained, among which 138 were identified as overlapping targets between BS and liver injury (Figure 3A). Furthermore, we used the STRING database to investigate the interactions of 138 targets. The analysis of centralities in the PPI network indicated 20 genes with the highest degree of centralities (Table S3).

Six modules were identified using MCODE analysis (Table S4). The highest score cluster 1 included 40 nodes and 478 edges with 24.51 scores (Figure 3B). The 10 core targets of 138 intersection targets were screened by the cytoHubba plugin (Figure 3C). HIF-1, VEGFA, STAT3 and AKT1 were closely related to the therapeutic effects of BS in NAFLD. A drug-compound-target-disease network for BS treatment of NAFLD was constructed using Cytoscape software (version 3.10.3) (Figure 3D). The integrative network suggests that the top five ingredients in terms of degree values were pedalitin, gondoic acid, catechin, camptothecin, and seamolin (Table S5), which may be the core components of BS against liver injury.

#### 3.3.2. KEGG and GO Enrichment Analysis of BSP for NAFLD

We performed the KEGG pathway enrichment analysis of potential target genes of BSP in NAFLD (*p* < 0.05) (Figure 3E and Table S6). These include regulation of the VEGF signaling pathway, HIF-1 signaling pathway, etc., by which the key targets are involved in NAFLD treatment. Additionally, using GO pathway enrichment analysis, we identified 163 BP terms, 101 MF terms, and 40 CC terms. We found the top 10 biological processes are closely related to NAFLD, including response to oxidative stress, response to decreased oxygen levels, etc. (Figure 3F and Table S7).

Network pharmacology results have shown that BS may improve NAFLD through its bioactive components, such as pedalitin and catechin. Notably, our previous studies have demonstrated that BSP exhibit the potential to ameliorate NAFLD. Furthermore, BSP shares structural similarities with pedalitin and catechin, including the presence of aromatic rings and hydroxyl groups, which confer similar bioactivities. Importantly, the extraction efficiency of BSP is significantly higher compared to other bioactive compounds in BS. Based on these findings, we hypothesize that BSP serves as the key bioactive component of BS. Future experiments will focus on investigating the specific mechanisms by which BSP improve NAFLD.

### 3.4. LC-MS Metabolomics Analysis

#### 3.4.1. Multivariate Statistical Analysis

**Figure 4:**
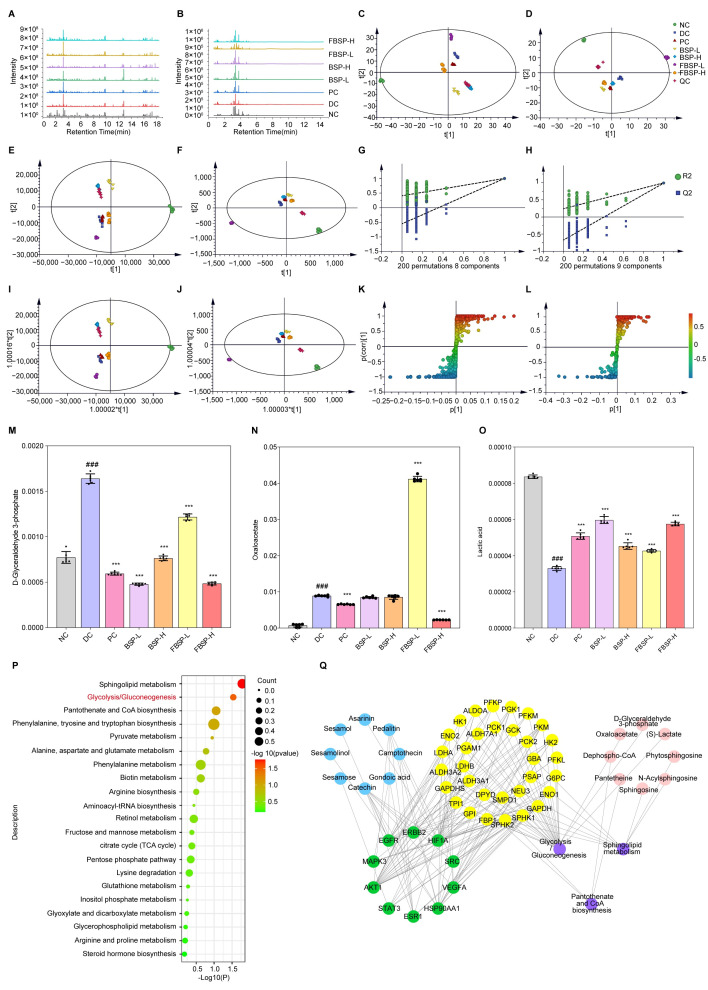
Multivariate data analysis from LC-MS. (**A**) LC-MS positive (ESI+) total ion chromatograms of different groups. (**B**) LC-MS negative (ESI-) total ion chromatograms of different groups. (**C**) PCA score plots in positive ion mode. (**D**) PCA score plots in negative ion mode. (**E**) PLS-DA score plots in positive ion mode. (**F**) PLS-DA score plots in negative ion mode. (**G**) PLS-DA model validation diagram in positive ion mode. (**H**) PLS-DA model validation diagram in negative ion mode. (**I**) OPLS-DA score plots collected from different groups in positive ion mode. (**J**) OPLS-DA score plots collected from different groups in negative ion mode. (**K**) S-plot of urine samples collected from NC and DC groups in positive ion mode. (**L**) S-plot of urine samples collected from NC and DC groups in negative ion mode. (**M**–**O**) Comparison of the relative intensity of D-glyceraldehyde-3-phosphate, oxaloacetate, and lactic acid in all the experimental groups. (**P**) Urine metabolic pathways result of BS on liver injury. (**Q**) PPI network diagram of “Active ingredient (blue), target of action (green), metabolic enzyme (yellow), metabolic pathway (purple), metabolite (red)” network diagram action targets. (### *p* < 0.001 compared with NC group, *** *p* < 0.001 compared with DC group).

The total ion current profile of urine samples is shown in Figure 4A,B. The results show that the metabolites in the urine samples of the mice in BSP-treated group had changed compared with that of NAFLD group, suggesting that BSP influences the metabolism of liver-injured mice and changes their physical condition.

PCA was carried out to determine whether BSP influenced the metabolic pattern of the liver-injured mice (Figure 4C,D). The FBSP-H groups were clearly separated from the DC group, suggesting that the high-dose group showed apparent anti-liver injury effects. PLS-DA is better suited to highlight the differences between groups (Figure 4E–H). The metabolic profiles in the BSP and FBSP treated groups deviated from the DC group and were close to the NC group, indicating that BSP and FBSP exerted potential protection from metabolic disturbances induced by liver injury.

#### 3.4.2. Differential Metabolites in Urine with Different Treatments

Based on OPLS-DA, we further investigated the potential biomarkers of BSP treatment for NAFLD (Figure 4I–L), and 39 significant differential metabolites were screened out (Table S8). The tendencies of the identified biomarkers related to liver injury varied. The 12 differentially abundant metabolites showed significant increases after BSP treatment (phytosphingosine, aminomalonic acid, chrysophanol, lactic acid, caffeic acid, guanidinosuccinic acid, 2-naphthylamine, L-arginine, D (−)-β-hydroxy butyric acid, D-biotin, sphingosine, and DL-2-aminoadipic acid), and four differential metabolites were significantly decreased (coenzyme Q4, D-glyceraldehyde-3-phosphate, ceramide, and oxaloacetate) in the BSP groups. These findings suggest that BSP may ameliorate NAFLD via these identified differential metabolites.

Based on the urinary metabolomics analysis, we found that ceramide, as an endotoxin substance, showed significantly higher levels in the NAFLD mice compared to the normal group. After BSP treatment, its level was significantly decreased (Figure S3). Ceramide has been linked to insulin resistance, oxidative stress, and inflammation [33,34,35], suggesting they play a role in the development of NAFLD. Studies have shown that the total concentration of ceramides in the liver is significantly increased in NAFLD [36,37,38,39], and elevated levels of C16-ceramides have been observed in the livers of HFD-fed mouse models [40]. Furthermore, increased plasma ceramide levels have been found in patients with NAFLD and type 2 diabetes [41,42]. These findings suggest that ceramides play a crucial role in the development of hepatic insulin resistance. In addition, ceramide levels are closely associated with gut barrier integrity. Studies have shown that the accumulation of ceramides impairs gut barrier function. Bacterial-derived sphingolipids can be absorbed by intestinal epithelial cells, processed, and transported to the liver through the portal vein [42]. Moreover, several bacterial toxins such as lipopolysaccharide (LPS), p-fimbriae, and the B-subunit of Shiga toxin increase ceramide levels through a TLR4-dependent response [43]. The studies indicate that NAFLD mice exhibit gut barrier damage and increased gut permeability, leading to elevated levels of endotoxin ceramides in urine. The intervention with BSP restored gut barrier injury and reduced urinary ceramide levels.

Investigation of the potential mechanisms underlying BSP-mediated improvement in NAFLD based on MetaboAnalyst analysis were performed (Figure 4P). The results show that the main metabolic pathway was glycolysis/gluconeogenesis (*p* = 0.029284, impact = 0.08591). The metabolites related to the pathway were D-glyceraldehyde-3-phosphate (Figure 4M), oxaloacetate (Figure 4N), and lactic acid (Figure 4O). This is in line with other studies which reported a marked increase in both glycolysis and gluconeogenesis in mice with NAFLD [44]. The product of glycolysis is pyruvate and the abnormal metabolism of pyruvate, along with an increase in lactate production, is an important indicator of liver damage. Hence, the metabolic pathways of glycolysis and gluconeogenesis may play an important role in the amelioration of mice with NAFLD by BSP and FBSP.

#### 3.4.3. Joint Pathway Analysis of Targets and Metabolites

To determine the mechanistic links between targets and metabolites, a component–target–metabolite–pathway network was constructed (Figure 4Q). HIF-1 and glycolysis/gluconeogenesis were selected as the most crucial therapeutic target and metabolic pathways. HIF1A, AKT1, MAPK3, and STAT3 are the key targets on this pathway. As previously reported, the HIF-1 signaling pathway is strongly associated with glycolytic degradation [45]. HIF1A activation affects glycolytic enzyme levels, including upregulation of hexokinase, pyruvate dehydrogenase, and lactate dehydrogenase. Brown adipocytes lacking HIF1A exhibit reduced glycolytic capacity, inhibited HIF-1 signaling pathway, and glucose uptake. Integrating network pharmacology with metabolomic analyses, we hypothesize that BSP regulates the HIF-1 signaling pathway, leading to changes in differential metabolites such as D-glyceraldehyde-3-phosphate, oxaloacetate, and lactic acid. Consequently, these alterations may influence the metabolic pathways of glycolysis/gluconeogenesis, thereby improving NAFLD in mice.

### 3.5. Gut Microbiota Analysis

#### 3.5.1. Intake of BSP Improved the Gut Microbiota Diversity

**Figure 5:**
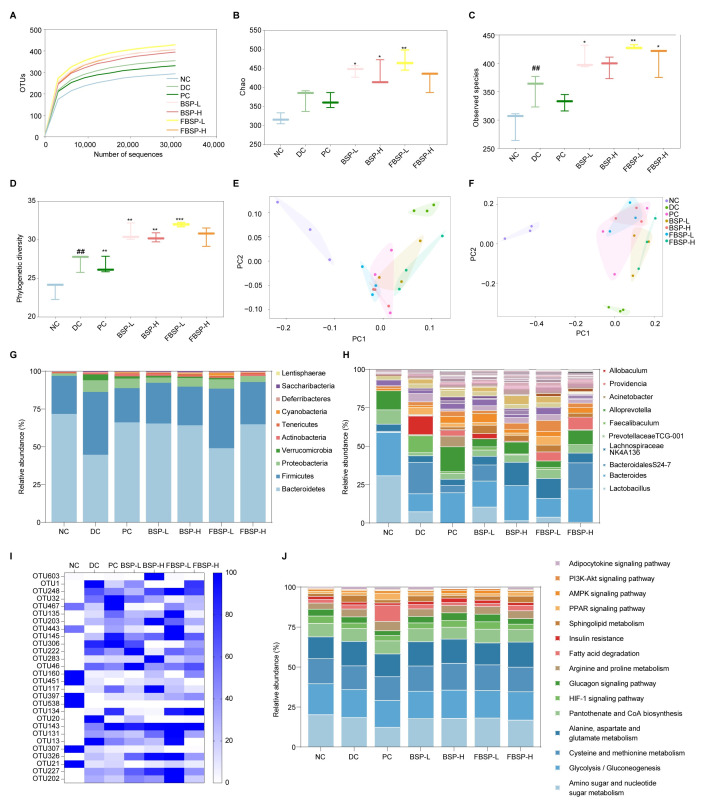
Overall structural modulation of gut microbiota. (**A**) Dilution curve of intestinal flora samples of mice. (**B**–**D**) Analysis of alpha diversity of mice gut microbiota (**B**): Observed species; (**C**): chao index; (**D**): Phylogenetic diversity). (**E**) PCA analysis based on OUT. (**F**) PCoA analysis based on OUT. (**G**) Relative abundance (%) of phylum in intestinal flora of mice. (**H**) Relative abundance (%) of genus in intestinal flora of mice. (**I**) Heat map of cluster analysis of OTUs distribution of different intestinal flora in mice. (**J**) Distribution and abundance of tertiary metabolic pathways in mouse intestinal bacteria. (## *p* < 0.01 compared with NC group, * *p* < 0.05, ** *p* < 0.01, *** *p* < 0.001 compared with DC group).

The OTU rarefaction curve tended towards a plateau at the end, indicating that the sequencing data volume was reasonable (Figure 5A), observed species (Figure 5B), chao index (Figure 5C) and Phylogenetic diversity (Figure 5D). The sparsity curve tending to be smooth indicates the rationality of sequencing data. Meanwhile, these indices were greatly increased in the BSP group compared to the DC group. We performed principal coordinate analysis (PCA) and principal coordinates analysis (PCoA) to visualize the beta diversity of microbial communities across different samples (Figure 5E,F). The gut microbiota composition in the BSP group is close to that in the NC group, indicating that BSP and FBSP have a regulatory effect on the gut microbiota of NAFLD mice.

#### 3.5.2. Intake of BSP Reversed the Gut Microbiota Community

At the phylum level, the NAFLD significantly increased the relative abundances of Firmicutes (42.05%) and Proteobacteria (7.89%), while it significantly decreased Bacteroidetes (45.17%) in the DC groups (*p* < 0.05). The administration of BSP reversed these changes (Figure 5G). At the genus level, the relative abundance of Bacteroides, Alloprevotella, and Prevotellaceae TCG-001 were significantly lower in the NAFLD group (*p* < 0.05), which was reversed by BSP intervention (Figure 5H). Based on the out level, differential bacterial species were screened (Figure 5I). A total of 28 OTUs with significant differences (*p* < 0.05) were selected. Considering the analysis results at the phylum and genus levels, BSP could potentially improve NAFLD through the enhancement of Lactobacillus, Bacteroides, Bifidobacterium, Akkermansia, and Allobaculum. In short, BSP and FBSP improves NAFLD in mice by regulating gut microbiota.

#### 3.5.3. Potential Functions of Gut Microbiota

Based on 16S rRNA and the KEGG database using the PICRUSt2, a total of 15 differential gut microbiota metabolic pathways (*p* < 0.05) were selected for functional prediction (Figure 5J). The differential gut microbiota genera were associated with the HIF-1 pathway (*p* = 1.27 × 10^−4^). Studies have shown that HIF1A is involved in gut barrier regulation [46]. HIF1A is involved in the regulation of gut microbiota, and studies have shown that HIF1A gene deficiency leads to dysbiosis of gut microbiota in mice. The research outcomes were consistent with network pharmacology and metabolomics studies.

Collectively, these multi-omics data confirmed that that BSP and FBSP can modulate the abundance of Lactobacillus, Bacteroides, Bifidobacterium, Akkermansia, and Allobaculum to regulate the HIF-1 signaling pathway, thereby improving the progression of NAFLD.

### 3.6. Liver Organoid Analysis

#### 3.6.1. Establishment and Characterization of Human Liver Organoids

**Figure 6:**
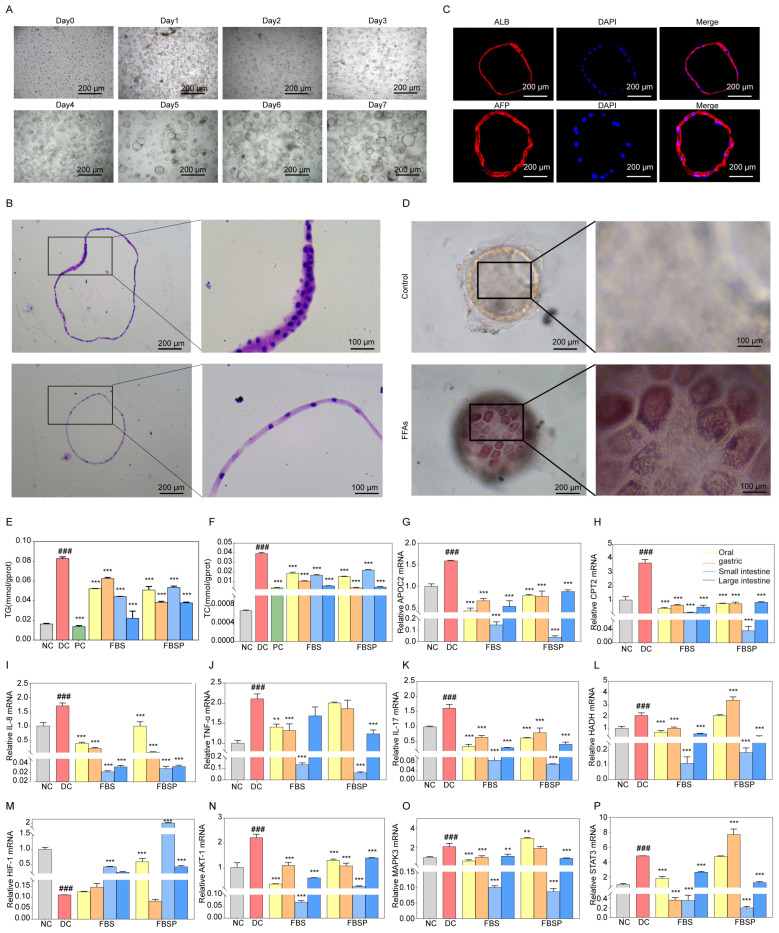
Mechanism research based on organoid level. (**A**) In vitro culture process of liver tissue adult cells. (**B**) H&E pathological observation of liver organoids. (**C**) Immunohistochemical staining of ALB and AFP were identified in liver organoids. (**D**) The production of droplets in liver organoids with FFAs treatment for 5 days by Oil Red O staining. (**E**,**F**) The intracellular lipid accumulation in liver organoids was assessed by the levels of triglyceride accumulation and total cholesterol. (**G**–**I**) Expression of APOC2, CPT2, and HADH mRNA in liver organoids. (**J**–**L**) Expression of TNF-α, IL-17, and IL-8 mRNA in liver organoids. (**M**–**P**) Expression of HIF1A, AKT1, MAPK3, and STAT3 mRNA in liver organoids. (### *p* < 0.001 compared with NC group, ** *p* < 0.01, *** *p* < 0.001 compared with DC group).

Liver organoid growth goes through different stages, such as preformation, growth, expansion, and maturation. The primary cells isolated from liver tissue aggregate in clusters (day 0), then the hepatocytes undergo rearrangement and enter a 3D culture mode (day 2), converging toward a spherical structure. The organoid appears to take on a spherical shape, during which folding and outgrowth occur (day 6). The growth morphology and volume stabilize (day 14), and the passaged growth can be used in subsequent experiments for 7 days (Figure 6A). We characterized these organoids using H&E staining (Figure 6B). H&E staining reveals that liver organoids are three-dimensional spherical structures. Immunofluorescence detection of hepatic progenitor cell and hepatocyte-specific marker of albumin (ALB) and alpha-Fetoprotein (AFP) expression indicates that the liver organoids possess efficient hepatic lineage differentiation and maturation (Figure 6C). Oil Red O staining revealed that liver organoids exposed to FFAs contained lipid droplets and underwent steatosis (Figure 6D). These data collectively validatethat the NAFLD model of liver organoid was successfully constructed.

#### 3.6.2. Effects of FBSP on Biomarkers of Lipid Metabolism and Inflammation

Both triglyceride (TG) and total cholesterol (TC) levels were significantly decreased in liver organoids from the FBSP treatment group (*p* < 0.05) (Figure 6E,F). These findings indicate that FBSP can reduce lipid accumulation caused by NAFLD. Additionally, the lipid metabolism-related genes, including apolipoprotein C2 (APOC2), carnitine palmitoyl transferase 2 (CPT2), and hydroxyacyl-CoA dehydrogenase (HADH), showed significantly higher expression levels in NAFLD organoids (*p* < 0.05) (Figure 6G–I). In addition, the levels of lipid metabolism-related genes were significantly downregulated in all treatment groups compared with the DC groups (*p* < 0.05). NAFLD is accompanied by chronic inflammatory responses, with pro-inflammatory cytokines including tumor necrosis factor-alpha (TNFα), interleukin-17 (IL-17), and interleukin-18 (IL-18). Compared to the DC group, these pro-inflammatory cytokines were significantly downregulated in the treatment group (*p* < 0.05) (Figure 6J–L).

#### 3.6.3. FBSP Modulates the Hepatic HIF-1 Pathway and Ameliorates NAFLD

Impairment of HIF-1 signaling pathway has been closely linked with NAFLD [47]. Compared with the NC group, the mRNA expression levels of hif-1alpha (HIF1A) in the liver organoids of the NAFLD group were significantly downregulated (*p* < 0.05), while the serine/threonine kinase 1 (AKT1), mitogen-activated protein kinase 3 (MAPK3), and Signal transducer and activator of transcription 3 (STAT3) were upregulated (Figure 6M–P). HIF1A mediates the hypoxia signaling pathway and may be involved in lipid degeneration and inflammation under hypoxic conditions [48]. Oxidative stress could be a critical factor contributing to the progression of NAFLD under hypoxic conditions. FBSP treatment significantly upregulated (*p* < 0.05) HIF1A levels in the oral, small, and large intestine groups, then inhibited the secretion of oxidative stress and pro-inflammatory factors to alleviate the development of NAFLD (Figure 6M).

AKT1 is an upstream target gene of the HIF-1 signaling pathway. It is involved in insulin-mediated glucose stabilization and hepatic lipid accumulation [49]. Additionally, AKT1 is involved in both inflammatory response and apoptotic signaling. Compared with the DC group, the AKT1 gene level was significantly downregulated in all treatment groups (*p* < 0.05) (Figure 6N). FBSP may inhibit inflammatory responses by regulating AKT1 expression levels, thereby improving NAFLD progression.

MAPK3 is involved in cell proliferation, division, apoptosis, and inflammatory response [50]. Studies have shown that activation of MAPK3 leads to triglyceride accumulation and promotes NAFLD progression [51]. Compared with the DC group, the MAPK3 gene expression level was significantly downregulated in fried FBSP treatment groups (*p* < 0.05), indicating that BSP may participate in the regulation of adipocyte differentiation and lipid metabolism, which is consistent with the results of lipid accumulation levels (Figure 6O). Meanwhile, FBSP may inhibit the activation of pro-inflammatory factors, such as IL-17, IL-8, and TNFα, by regulating MAPK3 expression, anti-inflammatory, and anti-immune effects, thereby improving NAFLD development.

STAT3 plays a crucial role in cellular functions, including inflammatory responses [52]. It has been shown that amelioration of liver injury through the STAT3 pathway reduces the secretion of oxidative stressors and inflammatory factors in liver tissue and affects lipid accumulation levels [53]. Compared with the DC group, the STAT3 gene expression level was significantly downregulated in the fried BSP groups (*p* < 0.05) (Figure 6P). FBSP may potentially alleviate the progression of NAFLD through inhibition of inflammatory factor expression, such as IL-17, IL-8, and TNFα, and inhibiting STAT3 expression levels, thereby reducing hepatocyte apoptosis, lipid accumulation, and inflammatory responses.

## 4. Discussion

NAFLD poses an increasing threat to human health and represents a new challenge in the field of modern medicine [54]. Our previous study indicated that BS ameliorates NAFLD, with BSP as a potential active ingredient, though the underlying mechanism remained elusive. To validate this hypothesis, we established a non-alcoholic fatty liver disease (NAFLD) model by combining a high-fat diet with streptozotocin [16,55,56,57], combined with multi-omics studies to investigate the mechanism by which BSP improves NAFLD. The high-fat diet induces metabolic disorders and hepatic lipid accumulation, while streptozotocin damages pancreatic β-cells, leading to diabetes and insulin resistance, thereby accelerating NAFLD progression. This model effectively mimics the insulin resistance and glucose metabolism disorders associated with NAFLD, which are consistent with the pathological characteristics observed in many NAFLD patients. In our study, BSP and FBSP were found to be able to significantly improve NAFLD, and FBSP showed stronger efficacy.

We found that HIF-1 is an important signaling pathway for BSP to ameliorate NAFLD through network pharmacology. HIF-1 is an upstream regulatory element in the body’s response to ischemia and hypoxia [58]. Accumulating evidence indicatesthat HIF-1 is involved in the occurrence and development of various liver diseases, its overexpression exerted a protective effect on hepatic ischemia-reperfusion injury, hypoxia/reoxygenation insult, and alleviating oxidative stress [59]. In metabolomics analysis, we identified gluconeogenesis/glycolysis was the major metabolic pathway involved in the amelioration of NAFLD by BSP. It has been shown that HIF1A activation is closely related to glycolysis and gluconeogenesis [60]. After HIF1A gene knockout in mice, the expression of glycolytic enzymes in brown adipocytes was reduced. Glucose uptake under hypoxic conditions in cells can be inhibited as a consequence of HIF-1 signaling pathway inhibition [61]. The above research further proves that BSP may ameliorate NAFLD through the HIF-1 signaling pathway and oxidative stress.

Consistent with prior reports, our metabolomic profiling reveals that NAFLD pathogenesis involves dysregulated glucose metabolism. Specifically, NAFLD mice exhibit hepatic glycogen depletion concomitant with upregulated glycolytic enzyme expression and excessive lactate generation—indicative of pathological glycolytic enhancement [62]. FBSP likely counteracts these changes by normalizing glycolytic activity, thereby restoring glycogen homeostasis and reducing lactate accumulation. Additionally, FBSP appears to suppress the aberrant gluconeogenic activity characteristic of NAFLD [63], attenuating futile cycling between glycolysis and gluconeogenesis while modulating tricarboxylic acid cycle metabolism. These convergent effects on glucose metabolism and oxidative stress reduction likely underlie FBSP’s capacity to ameliorate hepatic steatosis.

The gut microbiota has gained attention as a crucial factor influencing the occurrence and progression of NAFLD, and gut microbiota modulation offers a promising approach for the prevention or treatment of NAFLD [64]. Specifically, Bifidobacterium and Bacteroides have been implicated in the regulation of NAFLD-related metabolic disorders [65]. Thus, the role of BSP in ameliorating NAFLD may be related to increasing Bifidobacterial and Bacteroidales abundance, promoting bile acid metabolism, and maintaining hepatic metabolic homeostasis. Additionally, Lactobacillus can reduce cholesterol levels, thereby improving the progression of NAFLD [66]. Akkermansia is increasingly being recognized for its potential to improve NAFLD. The treatment of NAFLD mice with Akkermansia ameliorated their oxidative stress-induced intestinal apoptosis and remodeled gut microbiota composition [67]. Therefore, BSP ameliorated NAFLD by regulating the abundance of Lactobacillus, Bifidobacterium, Akkermansia, Allobaculum, and Bacteroidales.

We further identified through KEGG analysis that the HIF-1 signaling pathway was significantly enriched, which further supported the conclusions from network pharmacology and metabolomics studies, indicating that BSP may improve NAFLD via the HIF-1 signaling pathway. Recent studies have shown that there is intricate crosstalk between the gut and liver [68,69], which is mediated by the gut–liver axis and involves key microbial metabolites such as bile acids (BAs), short-chain fatty acids (SCFAs), and indoles. The gut–liver axis features a bidirectional communication network where microbially derived metabolites and immune signals influence liver function, while hepatic BAs regulate microbial composition and intestinal integrity. This suggests that it holds significant potential for the treatment of disease, with the capacity to become a major focus for the precise treatment of intestinal and liver disease. Furthermore, studies have indicated Hypoxia and HIF-1 as key regulators of gut microbiota and host interactions [70,71]. The HIF-1 signaling pathway interacts with gut microbiota to protect against liver injury. Therefore, we hypothesize that BSP may improve NAFLD through bidirectional regulation of gut microbiota and the HIF-1 signaling pathway. However, the temporal dynamics and mechanistic underpinnings of gut–liver interactions require further elucidation. Future studies employing antibiotic treatment and fecal microbiota transplantation (FMT) will be essential to dissect the causal relationships between intestinal microbiota alterations and hepatic signaling pathways.

Organoids are widely considered as genetically stable that can be programmed to virtually recapitulate certain biological, physiological, or pathophysiological features of original tissues or organs in vitro [72]. Therefore, we validated the mechanism by which FBSP improves NAFLD in liver organ models. In the liver organoids model, we observed HIF1A gene expression levels were increased after treatment with BSP groups. This is consistent with reported studies [73]. HIF1A is involved in preventing lipid accumulation. Lv et al. [74] demonstrated that liver-protecting activity targets liver injury by regulating the HIF-1 signaling pathway. Additionally, other studies have shown that dietary copper improves intestinal injury in alcoholic liver disease by modulating the HIF-1 oxidative stress signaling pathway in the gut [75]. Meanwhile, results show that FBSP may regulate the expression levels of the upstream genes STAT3, MAPK3, and AKT1 of the HIF-1 signaling pathway, thereby inhibiting oxidative stress, lipid accumulation, and secretion of inflammatory factors during NAFLD development. Although liver organoid models simulate human liver development processes and basic functions with high similarity and can well replicate hepatic cell metabolism and inflammatory responses, they still have limitations such as the lack of immune cells, hemodynamics, and inter-organ interactions. Therefore, the present study primarily reflects mechanisms at the hepatocyte level, and the complexity of the overall physiological environment requires further validation in in vivo models.

Prior studies have supported the critical regulatory role of the HIF-1 pathway in NAFLD. Animal model demonstrate that hepatic lipid accumulation induces perivenous hypoxia and HIF1A activation [76]. Hypoxia signals directly regulate hepatic lipid accumulation to improve metabolism. HIF-1 plays a crucial role in the regulation of peroxisomal lipid metabolism by activating the expression and nuclear accumulation of lipin1. Conversely, HIF1A deletion inhibits abnormal lipid accumulation by activating lipin1 expression, thereby suppressing peroxisomal fatty acid oxidation [73]. Furthermore, functional intervention experiments have been conducted to validate the critical role of HIF1A in NAFLD. Studies have found that overexpression of HIF1A can restore increased adipocyte volume, inflammation, and mitochondrial dysfunction in mice [77]. Compared to normal mice, liver-specific HIF1A knockout mice exhibit greater lipid accumulation [73]. These studies suggest that HIF1A, as the primary transcriptional regulator of the hypoxic response, regulates hepatic lipid metabolism and can serve as theoretical support for the conclusions of this study. However, the precise mechanisms linking HIF1A to NAFLD progression require further investigation. Future studies will employ targeted HIF1A inhibition to establish causality and dissect pathway-specific contributions. Importantly, we emphasize that this study provides new evidence implicating the HIF-1 pathway in FBSP-mediated NAFLD amelioration, rather than establishing it as the sole mechanism.

## 5. Conclusions

Our findings demonstrate the therapeutic potential of FBSP in NAFLD. Multi-omics analyses and liver organoid validation revealed that FBSP ameliorates oxidative stress, lipid metabolism, and levels of inflammatory factors through modulation of the HIF-1 signaling pathway, thereby attenating NAFLD progresssion. These results provide mechanistic insights into the hepatoprotective effects of BSP. This study has several limitations, including the need to validate other active ingredients identified by network pharmacology, elucidate the precise regulation of HIF-1 signaling pathways mediated by FBSP, and investigate the in vivo degradation process of FBSP.

## Data Availability

The original contributions presented in this study are included in the article/Supplementary Materials. Further inquiries can be directed to the corresponding authors.

## Supplementary Materials

The following supporting information can be downloaded at: https://www.mdpi.com/article/10.3390/antiox15020177/s1.

## Abbreviations

The following abbreviations are used in this manuscript:

APOC2	apolipoprotein C2	HFD	high-fat diet	NC	naive control
ALB	albumin	H&E	hematoxylin and eosin	NAFLD	non-alcoholic fatty liver disease
AFP	alpha-Fetoprotein	HADH	hydroxyacyl-CoA dehydrogenase	PCOA	principal coordinates analysis
AKT1	serine/threonine kinase 1	HIF1A	hif-1alpha	PCA	principal component analysis
BS	black sesame	IF	immunofluorescence	STZ	streptozotocin
BSP	black sesame pigment	IL-17	interleukin-17	SEM	mean ± standard error of the mean
CPT2	carnitine palmitoyl transferase 2	IL-18	interleukin-18	STAT3	Signal transducer and activator of transcription 3
FBSP	fired black sesame pigment	LC-MS	liquid chromatography-tandem mass spectrometry	TG	triglyceride
FFAs	free fatty acids	MCODE	molecular complex detection	TC	total cholesterol
FMT	fecal microbiota transplantation	MAPK3	mitogen-activated protein kinase 3	TNF-α	tumor necrosis factor-alpha
