# Supplementary material for: Black Sesame Pigment Ameliorates Non-Alcoholic Fatty Liver Disease via Modulation of the Gut–Liver Axis and HIF-1 Signaling Pathway
Source: Antioxidants (Basel). 2026 Jan 30;15(2):177. doi: 10.3390/antiox15020177 (PMC12938165; doi:10.3390/antiox15020177)
Supplement: Supplementary file 1 [file antioxidants-15-00177-s001.zip › antioxidants-4085031-supplementary.pdf]

Supporting figures

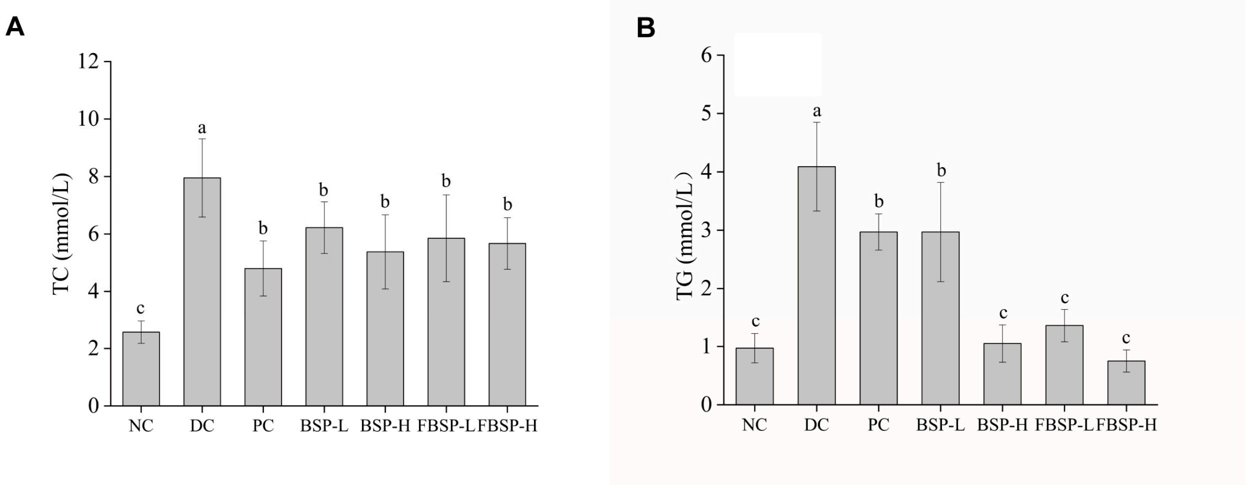

Figure S1. (A) Plasma total cholesterol content (mmol/L). (B) Plasma triglyceride content (mmol/L). (Different letters represent significant differences, $p < 0.05$)

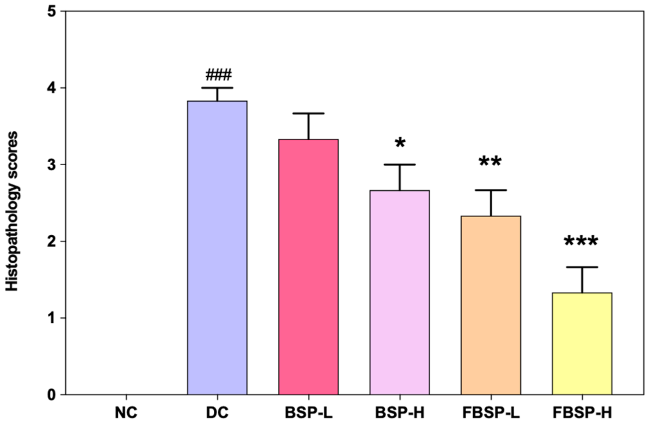

Figure S2. Histopathological scores for liver tissue in each group.

(#$p < 0.05$, ##$p < 0.01$, ###$p < 0.001$ compared with NC group, *$p < 0.05$, **$p < 0.01$, ***$p < 0.001$ compared with DC group)

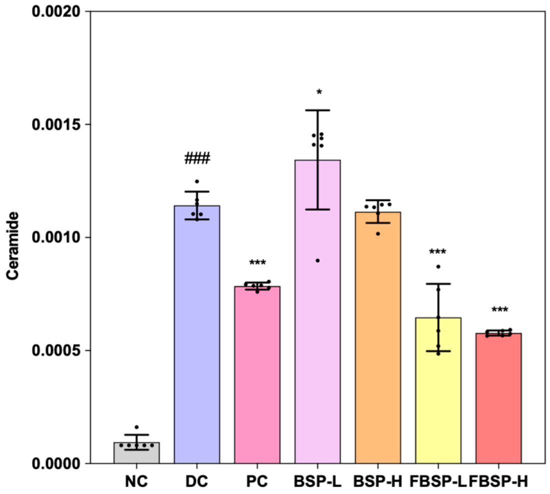

Figure S3. Ceramide content changes in urine samples of mice from different groups.

(#p < 0.05, ##p < 0.01, ###p < 0.001 compared with NC group, *p < 0.05, **p < 0.01, ***p < 0.001 compared with DC group)

Supplementary tables

Table S1. Primer sequences

Genes	Forward primer (5′-3′)	Reverse primer (3′-5′)
GAPDH	GAGTCAACGGATTTGGTCGT	GACAAGCTTCCCGTTCTCAG
APOC2	GCCCAGAACCTGTACGAGAAG	CATGGCTGCTGTGCTTTTGC
CPT2	CACCATGCACTACCAGGACA	GTTTGTCCAGAGCAACCAGC
IL-8	TCTGGCAACCCTAGTCTGCT	GCTTCCACATGTCCTCACAA
TNFα	ATCTACTCCCAGGTCCTCTTCA	TGGAAGACCCCTCCCAGATA
IL-17	GTCACTGCTACTGCTGCTGA	CGGGGGAAGTTCTTGTCCTC
HADH	TGTCGGACTGGATACTACGA	GATGGGCTGGGCTGATGTAA
HIF-1	ACTAGCCGAGGAAGAACTATGA	CTGAGGTTGGTTACTGTTGGTA
AKT-1	GGACAAGGACGGGCACATTA	CGACCGCACATCATCTCGTA
MAPK3	ACTACCTACAGTCTCTGCCCT	TCCTTAGGTAGGTCATCCAGC
STAT3	CTGCCCCATACCTGAAGACC	TCCTCACATGGGGGAGGTAG

Table S2 Physicochemical information of 11 potentially active components

Mol ID	Molecule Name	OB (%)	DL
MOL000433	Folic acid	68.96	0.71
MOL000492	Catechin	54.83	0.24
MOL005030	Gondoic acid	30.70	0.20
MOL009830	Camptothecin	61.04	0.81
MOL009834	Sesamolin	40.13	0.88
MOL009847	Pedalitin	34.02	0.31
MOL009849	Asarinin	31.57	0.83
MOL001595	Sesamol	66.34	0.03
MOL009836	Sesamoside	28.44	0.57
MOL009835	Sesamolinol	9.15	0.74
MOL009850	Sesamose	3.23	0.59

Table S3 Important 20 genes of intersection targets associated with BS and liver injury

Name	Degree	Neighborhood Connectivity	Topological Coefficient	Betweenness Centrality	Closeness Centrality	Clustering Coefficient
AKT1	84	29.11	0.22	0.09	0.72	0.28
VEGFA	74	31.59	0.24	0.04	0.68	0.35
SRC	72	31.32	0.23	0.06	0.67	0.34
EGFR	71	31.39	0.24	0.05	0.67	0.34
STAT3	68	32.24	0.24	0.03	0.65	0.38
MAPK3	68	31.63	0.24	0.05	0.66	0.34
HSP90AA1	63	32.79	0.24	0.04	0.64	0.38
ESR1	61	33.84	0.26	0.03	0.63	0.40
HIF1A	60	34.28	0.26	0.02	0.63	0.43
ERBB2	55	34.98	0.27	0.01	0.60	0.46
PTGS2	54	34.65	0.26	0.02	0.62	0.42
PPARG	52	31.83	0.24	0.04	0.62	0.34
MMP9	50	35.62	0.27	0.02	0.60	0.47
IL10	47	34.89	0.27	0.01	0.59	0.45
PPARA	41	30.78	0.23	0.04	0.59	0.31
CAT	40	34.75	0.26	0.03	0.58	0.40
MMP2	39	39.82	0.31	0.01	0.57	0.58
KDR	38	38.32	0.30	0.01	0.56	0.55
MAPK14	37	40.41	0.31	0.01	0.57	0.55

Table S4 Six clusters of the PPI network based on MCODE analysis

Cluster	Score	Nodes	Edges	Node IDs
1	24.51	40	478	HSP90AA1, HDAC1, ESR1, ESR2, PPARA, PLAU, PTK2, KIT, MMP9, FLT1, PPARG, MMP3, APP, CCNB1, SRC, MCL1, ERBB2, NOS2, PARP1, CAT, STAT3, VEGFA, PTGS2,KDR,NR3C1, IL10, MMP1, CCNA2, MAPK14, IGF1R, MAPK3, STAT1, CDK1, AKT1, PGR, DNMT1, TERT, AR, HIF1A, PLG
2	5.70	14	37	MMP14, GSK3B, ABCB1, TOP1, PIK3R1, MET, MMP2, ALK, ABCG2, CDK2, EGFR, HDAC6, FGFR1, PTPN11
3	5	5	10	SCD, NR1H3, RXRA, FABP1, FABP4
4	4.5	5	9	MAOA, OPRM1, MAOB, CYP2D6, SLC6A3
5	3	3	3	SLC6A4, ADORA2A, CNR1
6	3	5	6	CES1, CES2, PLA2G2A, F10, CYP3A4

Table S5 Physicochemical information of 11 components based on PPI network

compound	Degree	Betweenness Centrality	Closeness Centrality	Neighborhood Connectivity	Topological Coefficient
Pedalitin	68.00	0.11	0.49	2.97	0.18
Gondoic acid	47.00	0.06	0.43	2.91	0.17
Catechin	45.00	0.05	0.42	3.37	0.22
Camptothecin	22.00	0.01	0.38	4.14	0.29
Sesamolin	15.00	0.01	0.37	3.60	0.24
Asarinin	9.00	0.00	0.36	5.00	0.36
Folic acid	9.00	0.00	0.36	4.00	0.27
Sesamol	8.00	0.00	0.35	5.63	0.42
Sesamose	5.00	0.00	0.35	4.00	0.27
Sesamoside	2.00	0.00	0.34	7.00	0.55
Sesamolinol	1.00	0.00	0.34	11.00	0.00

Table S6 KEGG pathway enrichment information

Description	*p* value	Genes	Count
Proteoglycans in cancer	3.28974E-13	AKT1/ERBB2/ESR1/HIF1A/IGF1R/KDR/MAPK14/MAPK3/MMP9/PLAU/PTK2/SRC/STAT3/VEGFA	14
Endocrine resistance	2.43244E-11	AKT1/ERBB2/ESR1/ESR2/IGF1R/MAPK14/MAPK3/MMP9/PTK2/SRC	10
VEGF signaling pathway	2.90798E-10	AKT1/KDR/MAPK14/MAPK3/PTGS/PTK2/SRC/VEGFA	8
Prostate cancer	6.49084E-10	AKT1/AR/ERBB2/HSP90AA1/IGF1R/MAPK3/MMP3/MMP9/PLAU	9
Progesterone-mediated oocyte maturation	1.02247E-09	AKT1/CCNA2/CCNB1/CDK1/HSP90AA1/IGF1R/MAPK14/MAPK3/PGR	9
Prolactin signaling pathway	1.1912E-09	AKT1/ESR1/ESR2/MAPK14/MAPK3/SRC/STAT1/STAT3	8
HIF-1 signaling pathway	1.85784E-09	AKT1/ERBB2/FLT1/HIF1A/IGF1R/MAPK3/NOS2/STAT3/VEGFA	9
EGFR tyrosine kinase inhibitor resistance	3.18966E-09	AKT1/ERBB2/IGF1R/KDR/MAPK3/SRC/STAT3/VEGFA	8
Chemical carcinogenesis - receptor activation	3.31202E-09	AKT1/AR/ESR1/ESR2/HSP90AA1/MAPK3/PGR/PPARA/SRC/STAT3/VEGFA	11
Lipid and atherosclerosis	3.84165E-09	AKT1/HSP90AA1/MAPK14/MAPK3/MMP1/MMP3/MMP9/PPARG/PTK2/SRC/STAT3	11

Table S7 GO pathway enrichment information

Ontology	Description	*p* value	Gene ID	Count
BP	response to peptide	4.7466E-17	AKT1/APP/CAT/CCNA2/IGF1R/IL10/MAPK14/MMP3/MMP9/PARP1/PPARA/PPARG/PTGS2/PTK2/SRC/STAT1/STAT3	17
BP	reproductive structure development	5.0883E-15	AKT1/AR/ESR1/HIF1A/IL10/KDR/KIT/MAPK14/PGR/PLG/PPARG/PTGS2/PTK2/SRC/VEGFA	15
BP	reproductive system development	5.63164E-15	AKT1/AR/ESR1/HIF1A/IL10/KDR/KIT/MAPK14/PGR/PLG/PPARG/PTGS2/PTK2/SRC/VEGFA	15
BP	muscle cell proliferation	4.06675E-14	AKT1/CDK1/DNMT1/HDAC1/IGF1R/IL10/MAPK14/MMP9/PPARG/PTGS2/STAT1/TERT	12
BP	response to peptide hormone	5.00142E-14	AKT1/CAT/CCNA2/IGF1R/IL10/MAPK14/PARP1/PPARA/PPARG/PTGS2/PTK2/SRC/STAT1/STAT3	14
BP	response to oxidative stress	1.29035E-13	AKT1/APP/CAT/CDK1/HIF1A/IL10/MAPK3/MCL1/MMP3/MMP9/PARP1/PTGS2/SRC/STAT1	14
BP	epithelial cell proliferation	1.76162E-13	AKT1/AR/ERBB2/ESR1/FLT1/HIF1A/IL10/KDR/KIT/PGR/PPARG/STAT1/STAT3/VEGFA	14
BP	positive regulation of smooth muscle cell proliferation	3.50344E-13	AKT1/DNMT1/HDAC1/IGF1R/IL10/MMP9/PTGS2/STAT1/TERT	9
BP	response to decreased oxygen levels	7.89104E-13	AKT1/CAT/CCNA2/HIF1A/NOS2/PLAU/PPARA/PPARG/PTGS2/SRC/TERT/VEGFA	12
BP	regulation of smooth muscle cell proliferation	1.87839E-12	AKT1/DNMT1/HDAC1/IGF1R/IL10/MMP9/PPARG/PTGS2/STAT1/TERT	10
CC	caveola	2.32143E-05	IGF1R/MAPK3/PTGS2/SRC	4
CC	secretory granule lumen	4.5061E-05	APP/CAT/HSP90AA1/MAPK14/PLG/VEGFA	6
CC	cytoplasmic vesicle lumen	4.74469E-05	APP/CAT/HSP90AA1/MAPK14/PLG/VEGFA	6
CC	membrane raft	4.82644E-05	APP/IGF1R/KDR/MAPK3/PTGS2/SRC	6
CC	transcription regulator complex	4.8789E-05	ESR1/HDAC1/HIF1A/PARP1/PPARG/STAT1/STAT3	7
CC	vesicle lumen	4.90931E-05	APP/CAT/HSP90AA1/MAPK14/PLG/VEGFA	6
CC	membrane microdomain	4.90931E-05	APP/IGF1R/KDR/MAPK3/PTGS2/SRC	6
CC	plasma membrane raft	8.16547E-05	IGF1R/MAPK3/PTGS2/SRC	4
CC	protein kinase complex	8.74165E-05	CCNA2/CCNB1/CDK1/IGF1R	4
CC	ficolin-1-rich granule lumen	0.000117066	CAT/HSP90AA1/MAPK14/MMP9	4
MF	nuclear receptor activity	1.65445E-13	AR/ESR1/ESR2/NR3C1/PGR/PPARA/PPARG/STAT3	8
MF	ligand-activated transcription factor activity	1.65445E-13	AR/ESR1/ESR2/NR3C1/PGR/PPARA/PPARG/STAT3	8
MF	transcription coregulator binding	1.37712E-12	AR/ESR1/HDAC1/HIF1A/PGR/PPARA/PPARG/STAT1/TERT	9
MF	transcription coactivator binding	2.32324E-12	AR/ESR1/HIF1A/PGR/PPARA/STAT1/TERT	7
MF	RNA polymerase II-specific DNA-binding transcription factor binding	1.33245E-10	AR/ESR1/HDAC1/HIF1A/MAPK14/PARP1/PPARA/PPARG/SRC/STAT1/STAT3	11
MF	nuclear steroid receptor activity	1.54071E-09	ESR1/ESR2/NR3C1/PGR/PPARA	5
MF	protein serine/threonine/tyrosine kinase activity	1.8316E-09	AKT1/CDK1/ERBB2/FLT1/IGF1R/KDR/KIT/MAPK14/MAPK3/PTK2/SRC	11
MF	DNA-binding transcription factor binding	3.16709E-09	AR/ESR1/HDAC1/HIF1A/MAPK14/PARP1/PPARA/PPARG/SRC/STAT1/STAT3	11
MF	protein tyrosine kinase activity	1.48559E-08	ERBB2/FLT1/IGF1R/KDR/KIT/PTK2/SRC	7
MF	phosphatase binding	1.73637E-07	ERBB2/MAPK14/MAPK3/PPARA/PTK2/STAT1/STAT3	7

Table S8 Differential metabolite associated with liver injury in urine detected by LC-MS

NO.	Metabolites	RT (min)	m/z	Formula	VIP	*p* value	HMDB	Scan mode
1	beta-D-Glucosamine	0.65	179.07	$C_6H_{13}NO_5$	1.03	5.61E-08	0030091	+
2	DL-2-Aminooctanoic acid	0.80	159.12	$C_8H_{17}NO_2$	1.03	5.12E-08	0000991	+
3	DL-2-Aminoadipic acid	1.09	161.06	$C_6H_{11}NO_4$	1.03	6.44E-10	0000510	+
4	L-(-)Sorbose	1.62	178.08	$C_6H_{12}O_6$	1.02	6.16E-07	0001266	+
5	Erythrose	1.76	120.05	$C_4H_8O_4$	1.03	7.76E-12	0002649	+
6	L-Phenylalanine	1.90	165.07	$C_9H_{11}NO_2$	1.03	3.85E-09	0000159	+
7	D(-)-β-hydroxy butyric acid	2.46	104.05	$C_4H_9NO_3$	1.03	1.26E-12	0000011	+
8	Pantetheine	2.52	278.12	$C_{11}H_{22}N_2O_4S$	1.03	1.31E-07	0003426	+
9	Salicyluric acid	2.87	195.05	$C_9H_9NO_4$	1.03	1.07E-08	0000840	+
10	L-Arginine	3.16	174.11	$C_6H_{14}N_4O_2$	1.03	1.26E-12	0000517	+
11	O-Phosphoryl-L-homoserine	3.22	199.03	$C_4H_{10}NO_6P$	1.03	2.10E-07	0003484	+
12	Chloroacetic acid	3.24	93.91	$C_2H_3ClO_2$	1.03	2.29E-08	0031331	-
13	2-Naphthylamine	3.30	143.07	$C_{16}H_{13}N$	1.03	2.06E-13	0032865	+
14	Coumarin	3.46	146.04	$C_9H_6O_2$	1.03	3.09E-10	0001218	+
15	Xanthurenic acid	3.47	205.03	$C_{10}H_7NO_4$	1.03	1.03E-10	0000881	+
16	Oxaloacetate	3.50	131.90	$C_4H_4O_5$	1.03	8.09E-10	0000223	-
17	Ceramide (d18:1/12:0)	3.58	481.45	$C_{48}H_{93}NO_6$	1.03	2.31E-10	0004947	-
18	Caffeic Acid	3.63	180.05	C9H8O4	1.03	3.80E-15	0001964	+
19	LysoPE(0:0/24:6)	3.67	553.35	C29H48NO7P	1.03	2.72E-09	0011499	-
20	LysoPC(22:1)	3.78	577.38	C30H60NO7P	1.01	5.56E-07	0010399	-
21	Guanidinosuccinic Acid	4.76	175.06	C5H9N3O4	1.03	3.55E-14	0003157	+
22	Lactic acid	4.76	90.04	C3H6O3	1.03	1.65E-15	0000190	+
23	Aminomalonic acid	5.01	119.03	C3H5NO4	1.03	3.49E-16	0001147	+
24	D-Glyceraldehyde-3-phosphate	5.06	169.86	C3H7O6P	1.02	4.20E-10	0001112	-
25	Pyroglutamic acid	5.19	129.05	C5H7NO3	1.03	1.79E-11	0000267	+
26	Retinoic acid	5.70	300.20	C20H28O2	1.03	4.42E-10	0001852	+
27	D-Biotin	5.90	244.08	C10H16N2O3S	1.03	3.34E-13	0000030	+
28	Mesaconic acid	6.03	130.03	C5H6O4	1.03	9.76E-11	0000749	+
29	L-Nicotine	6.07	162.12	C10H14N2	1.02	5.33E-09	0001934	+
30	2-Keto-glutaramic acid	6.10	145.04	C5H7NO4	1.03	3.86E-10	0001552	+
31	Coenzyme Q4	6.14	454.32	C29H42O4	1.03	3.57E-11	0006710	+
32	Chrysophanol	6.80	254.05	C15H10O4	1.03	8.42E-16	0030670	+
33	Glycitein	7.02	284.07	C16H12O5	1.03	1.53E-10	0005781	+
34	Cortisol	7.91	362.21	C21H30O5	1.03	1.20E-09	0000063	+
35	Sphingosine	8.67	299.28	C18H37NO2	1.03	7.48E-12	0000252	+
36	Phytosphingosine	10.07	317.29	C18H39NO3	1.03	9.26E-17	0004610	+
37	Estriol	10.11	288.17	C18H24O3	1.03	6.13E-08	0000153	+
38	Stearamide	17.25	283.29	C18H37NO	1.01	2.49E-08	0034146	+
39	Dihydroxyfumarate	17.29	148.01	C4H4O6	1.00	3.44E-06	0002050	+
